# Application of an economic calculator to determine the cost of porcine reproductive and respiratory syndrome at farm-level in 21 pig herds in Germany

**DOI:** 10.1186/s40813-020-00183-x

**Published:** 2021-01-04

**Authors:** C. Renken, C. Nathues, H. Swam, K. Fiebig, C. Weiss, M. Eddicks, M. Ritzmann, H. Nathues

**Affiliations:** 1 Clinic for Swine at the Centre for Clinical Veterinary Medicine, LMU Munich, Sonnenstrasse 16, 85764 Oberschleissheim, Germany; 2 Veterinary Public Health Institute, Vetsuisse Faculty, University of Bern, Schwarzenburgstrasse 155, 3097 Liebefeld, Switzerland; 3 Center for Diagnostic Solutions, MSD AH Boxmeer, Wim de Körverstraat 35, Boxmeer, 5831 AN The Netherlands; 4 MSD Animal Health, Feldstrasse 1A, 85716 Unterschleissheim, Germany; 5 Clinic for Swine, Vetsuisse Faculty, University of Bern, Bremgartenstrasse 109a, 3012 Bern, Switzerland

**Keywords:** PPRSV endemic infection, Economic loss, Cost simulation tool, PRRS control

## Abstract

**Background:**

Porcine Reproductive and Respiratory Syndrome Virus (PRRSV) continues to be a major economic issue for the swine industry worldwide, not only due to acute outbreaks but also endemic infections. PRRS disease severity and consequently financial losses can vary greatly between endemically infected farms and estimation of damage is challenging. This study aimed to assess the economic effect of PRRS in a systematic way at individual farm-level for endemically infected herds, using a PRRS cost simulation tool. In total 21 German sow herds with endemic PRRSV infection were investigated. Data on health and production performance, farm management and environment to be fed into the calculator was collected on each farm, and blood samples taken to confirm the PRRSV status.

**Results:**

All study farms experienced a significant loss attributable to PRRS. The median farm budget across all farms was − 31 € per sow and year, compared to a median simulated farm budget of 248 € if these farms had been PRRSV negative. The median total loss attributable to PRRS was 74,181 € per farm per year, corresponding to a median total loss per sow and year of 255 €. The impact of PRRS on farm profits was − 19.1% on average and − 41% in the worst case.

**Conclusions:**

The calculated losses give a good hint of the economic damage due to PRRS for the pig industry. Even in endemically infected farms, farmers face a non-negligible damage and profit from a concerted PRRS control. The calculator has proven itself in the field to render a valid estimation of losses due to PRRS in endemically infected farms.

## Background

First isolated in the Netherlands, Porcine Reproductive and Respiratory Syndrome Virus (PRRSV) impacts modern pig production worldwide, and continues to be a major economic issue for the swine industry [1, 2]. Various economic analyses confirmed the vast financial impact of clinical outbreaks of Porcine Reproductive & Respiratory Syndrome (PRRS) on pig production [3–7]. While in 2005, PRRS outbreaks in the US caused financial losses of approximately $560 million per year, a more recent calculation in 2013 estimates the costs of productivity losses in the US due to PRRS to be as high as $664 million per year [3, 4].

The epidemic form in acute outbreaks of PRRS is characterized by massive reproductive disorders in sows, perinatal losses and respiratory distress in piglets [8, 9]. The main impact in breeding units arises from a reduction in the number of weaned piglets and a reduced farrowing rate, while in nursery and finishing pigs it arises from an increase of morbidity and mortality rates, reduction in feed efficiency and growth rates and therefore an increase in unmarketable pigs [3]. Furthermore, increased expenses associated with prevention and treatment of secondary infections have an indirect impact on the cost of production [3, 6]. In addition to the genetic variation of different PRRSV isolates, various management practices on-farm can influence the clinical signs of a PRRS outbreak [6]. The duration and impact of a PRRS outbreak can be highly variable among farms [3, 10]. Besides farm size further factors related to farm internal processes like pig flow, biosecurity or hygiene were proven to be associated to clinical presentation of PRRS in individual farms [10, 11].

Even though study results indicate that after infection, most pigs clear the PRRSV within 3 to 5 months, epidemic infection leads to individuals remaining persistently infected for several months, up to 251 days [12–14]. Summarized in a study in 2016, it can be assumed even if contagiousness decreases over time; transmission of PRRSV is possible under natural conditions up to 3 months after infection in horizontally infected pigs, and even longer in congenitally infected animals [15]. Transmission by carrier animals can lead to long-term herd infection. In 2000, a mathematical simulation estimated that the average time for type 1 PPRSV to fade-out was about 6 years in a closed herd of 115 sows, whereas it took 80 years in a herd of 230 sows [9]. Particularly in large herds with multiple cohorts a slow and uneven spread of PRRSV and consequently a long duration of infection can occur [10]. Besides its ability to persist in animals and its high transmissibility, critical limitations of PRRSV control and eradication arise from the high risk of virus introduction and the genetic variability [16, 17].

In contrast to an acute PRRS outbreak the endemic phase of the disease causes fewer clinical signs [5]. The economic impact of PRRS in the breeding herd is not confined to the acute phase of an outbreak [7]. In endemically infected herds, where according to Hippocrates ‘endemic’ means that some forms of sickness is always present in a population [18], reproductive performance can still be diminished and PRRSV can be present in the nursery for more than 2.5 years after an acute outbreak and increase susceptibility to bacterial infections [7, 19]. Summarized by the same authors, costs attributable to a persistent PRRSV infection of growing pigs range between US$ 6.25–15.25 per pig [7]. In 2012, a Dutch group compared data of 9 PRRS affected breeding or nucleus herds 18 weeks after an outbreak and described great variation in financial losses ranging from US$ 3–160 per sow and year, including costs for different control strategies [5].

These studies indicate that PRRS disease severity and consequently financial losses can vary greatly between endemically infected farms and estimation of damage is challenging. Although many calculations on the impact of PRRS are available, most of them are general estimates at industry level, derived from anecdotal case reports, or consider just an epidemic period on farm [3–5]. The present study assesses the economic effect of PRRS in a systematic way at individual farm-level for endemically infected herds by using an economic simulation model [20]. Besides taking different disease severity levels into account, the model is easily adaptable to different farm settings and can consider the national market situation. The simulation tool should serve as a farm-level support tool for farmers and veterinarians, to assess farm profitability in the presence of PRRS and to illustrate farmers the costs of PRRS.

Based on this awareness of farm-individual financial losses due to PRRS, various control strategies are worth considering for farmers and veterinarians. Different control strategies such as vaccination or eradication are described to be effective in numerous case-reports and calculations [16, 21, 22]. Practitioners and farmers frequently ask themselves whether this also applies to their own particular case since implementing control strategies is a major investment for a farmer and it is not necessarily the most effective measure that turns out to be the most economically efficient one [23]. The presented simulation tool investigates in a second step the farm-individual efficiency of different control strategies via scenario analysis. This can simplify consulting farmers and objectify the decision which control strategy fits best in a certain farm.

The aim of the present study was to apply this simulation tool for veterinarians under field conditions and evaluate its practical applicability in the field by, in a first step, estimating losses due to an endemic PRRSV infection.

## Results

### Farm data

Data from all 21 farms were collected during individual interviews that lasted between 20 min and 120 min. All farmers and farm workers, respectively, provided detailed information about the breeding part of their farm, which included production data and economic data of the last 12 months prior to the interview. In four farms, specific production data and economic data for the nursery part were not available, and in three farms no data was provided for the fattening part. Within the economic calculator determining the cost of PRRS and the economic efficiency of intervention strategies for the individual herd, default values for the German pig industry were used [20] wherever no specific value was entered into the system.

The 21 herds in this study were accommodating six different breeds: crossbreed of Large White and Landrace (*n* = 5), Danzucht (*n* = 5), Danish Landrace (*n* = 4), BHZP (*n* = 3), Topigs (*n* = 2), PIC (*n* = 1) and unknown (*n* = 1). Piglets were produced in batches every 1 to 5 weeks: 1-week-rhythm (*n* = 3), 2-weeks-rhythm (*n* = 2), 3-weeks-rhythm (*n* = 10), 4-weeks-rhythm (*n* = 5) and 5-weeks-rhythm (*n* = 1). The suckling period was either 3 weeks (*n* = 7) or 4 weeks (*n* = 14). Other data assessed for the breeding, nursery and fattening part is summarized in Tables 1, 2 and 3.

**Table 1:** Farm data and economic data describing the breeding part of 21 herds endemically infected with Porcine Reproductive and Respiratory Syndrome Virus (PRRSV) and suffering from corresponding disease in sows, weaners, growers or finishing pigs

***Breeding***	*Median*	*Min.*	*Max.*	*Mean*	*SD*
**Farm data**					
Number of working sows in the farm per year (n)	330	150	1200	355	215
Production rhythm (weeks)	3	1	5	2.9	1.07
Length of suckling period (weeks)	4	3	4	3.7	0.5
Replacement rate per year (%)	39.5	26.9	50	39.3	7.2
Feed consumption (gestation) / sow from insemination to farrowing (kg)	300	275	418	316.8	42.5
Feed consumption (lactation) per sow during suckling period (kg)	160	90	260	167	43.8
*Return-to-estrus rate (%)*^a^	8.4	3	20	8.9	4.3
*Abortions (%)*^a^	1.6	0.3	15	2.5	3.1
*Piglets born alive per sow per litter (n)*^a^	15.7	11.7	18.4	15.4	1.5
*Preweaning mortality (%)*^a^	13.6	10	19	13.7	2.5
*Weight of suckling pigs at weaning (kg)*^a^	7.4	5.3	9.5	7.1	1.1
**Economic data**					
Price per 1000 kg gestation feed (€)	232.25	211.00	380.00	245.29	40.61
Price per 1000 kg lactation feed (€)	278.00	258.00	420.00	287.18	39.40
Veterinary cost per sow per year incl. vaccination costs (€)	193.60	80.00	250.00	177.02	47.81
Price per dose PRRS vaccination (sow) incl. labour (€)	1.56	1.00	2.40	1.60	0.32
Price per dose PRRS vaccination (piglet) incl. labour (€)	1.49	0.33	1.81	1.34	0.44
Costs for a replacement gilt (€)	350.00	250.00	400.00	348.40	39.46
Price per sow slaughtered / replaced (€)	240.00	191.00	500.00	250.69	69.91
Price per semen dose (€)	4.00	2.20	6.00	4.21	1.16
Energy cost per sow and year (€)	62.50	40.00	196.00	73.57	42.73
Labour cost per sow and year (€)	250.00	131.25	438.00	244.48	83.13
Building cost per sow and year (€)	100.00	25.00	200.00	103.90	51.38
Equipment cost per sow and year (€)	10.00	5.00	200.00	44.31	77.20
Inspection, Levy and Insurance cost per sow and year (€)	15.75	1.00	47.00	18.33	13.17
Transport costs for slaughter sows per sow (€)	4.40	0.00	17.00	4.56	4.55
Any other variable cost per sow and year (€)	0.00	0.00	0.00	0.00	0.00
Any other fix cost per sow and year (€)	0.00	0.00	155.80	9.56	34.40

^a^Parameters in italic are the ones potentially altered if the farm is affected by PRRS

**Table 2:** Farm data and economic data describing the nursery part of 17 herds endemically infected with PRRSV and suffering from corresponding disease in sows, weaners, growers or finishing pigs

***Nursery***	*Median*	*Min.*	*Max.*	*Mean*	*SD*
**Farm data**					
Time, weaners spend in the nursery (days)	53	35	100	55.3	14.9
Downtime between turns in the nursery (days)	5	0	14	4.9	3.6
Weight of weaners, when sold/moved to fattening (kg)	29.3	22	50	30.7	6.4
*% of weaners clinically affected by PRRS (including those later dying)*^a^	10	0	30	9.1	7.4
*Mortality in weaners (%)*^a^	2.5	1.5	6	2.9	1.2
**Economic data**					
Price per kg live weight of a weaner sold (€)	2.15	1.50	2.90	2.23	0.52
Total veterinary cost per weaner produced (€)	2.25	0.00	17.50	3.40	4.48
Price per 1000 kg piglet feed (€)	320.00	279.00	460.00	336.57	49.13
Energy cost per weaner produced (€)	0.75	0.00	62.50	8.71	21.75
Labour cost per weaner produced (€)	1.00	1.00	3.50	1.57	0.93
Building cost per weaner produced (€)	2.75	0.25	7.50	3.15	2.45
Equipment cost per weaner produced (€)	0.56	0.10	1.00	0.58	0.42
Inspection, Levy & Insurance cost per weaner produced (€)	0.33	0.23	0.61	0.37	0.18
Any other variable cost per weaner produced (€)	0.00	0.00	0.00	0.00	0.00
Any other fix cost per weaner produced (€)	0.00	0.00	0.00	0.00	0.00

^a^Parameters in italic are the ones potentially altered if the farm affected by PRRS

**Table 3:** Farm data and economic data describing the fattening part of 18 herds endemically infected with PRRSV and suffering from corresponding disease in sows, weaners, growers or finishing pigs

***Fattening***	*Median*	*Min.*	*Max.*	*Mean*	*SD*
**Farm data**					
Duration of fattening until pigs go to slaughter (days)	112	88	190	115.6	21.4
Downtime between turns in the fattening units (days)	5	2	15	5.3	3.5
Weight of fattening pigs at slaughter (kg)	120	118	130	121.5	3.7
*% fatteners clinically affected by PRRS (including those later dying)*^a^	10	0	50	13.9	13.7
*Mortality in fatteners (%)*^a^	2	1	4	2.2	1
**Economic data**					
Price per kg live weight of a fattener sold (€)	1.44	1.18	3.80	1.64	0.78
Total veterinary cost per fattening pig (€)	0.60	0.11	1.70	0.84	0.55
Fatteners’ feed price per 1000 kg	245.00	210.00	390.00	259.96	57.85
Energy cost per pig produced (€)	3.69	1.00	5.00	3.49	1.60
Transport cost per kg live weight slaughter pig (€)	0.027	0.00	0.05	0.023	0.016
Labour cost per pig produced (€)	7.30	3.00	9.00	5.94	2.79
Building cost per pig produced (€)	6.00	3.00	18.75	9.68	6.50
Equipment cost per pig produced (€)	2.00	0.10	2.44	1.71	0.92
Inspection, Levy and Insurance cost per pig produced (€)	0.80	0.10	2.00	0.90	0.70
Any other variable cost per pig produced (€)	0.00	0.00	0.90	0.04	0.20
Any other fix cost per pig produced (€)	0.00	0.00	10.00	0.57	2.19

^a^Parameters in italic are the ones potentially altered if the farm affected by PRRS

Overall, 19 farmers used (modified) live vaccines against PRRSV either in sows only (*n* = 7) or in sows and piglets (*n* = 12) in order to prevent disease in their herd. Mass vaccination of all sows was conducted every 3 months (*n* = 4) or every 4 months (*n* = 8). The sows in the remaining herds were vaccinated at 6 days *post-partum* and 60 days of pregnancy (*n* = 3) or at various time points (*n* = 2); two herds did not indicate a specific scheme.

All study herds showed significant alterations in two or more of the eight health and performance parameters that PRRS is assumed to have an impact on, according to Nathues and others [20] (parameters in italic in Tables 1, 2 and 3): return-to-estrus rate (> 10%, where 10% is the upper limit for a farm considered PRRSV negative); abortion rate (> 2%); average piglets born alive (depending on genetics, < 12.7 on average); pre-weaning mortality (> 11%); mortality in weaners (> 3%) and fatteners (1.5%); PRRS morbidity in weaners and fatteners.

### Laboratory data

Overall, 1507 blood samples were collected during the study, resulting in a median number of 73 per herd (Min: 67, Max: 74).

**Fig. 1:**
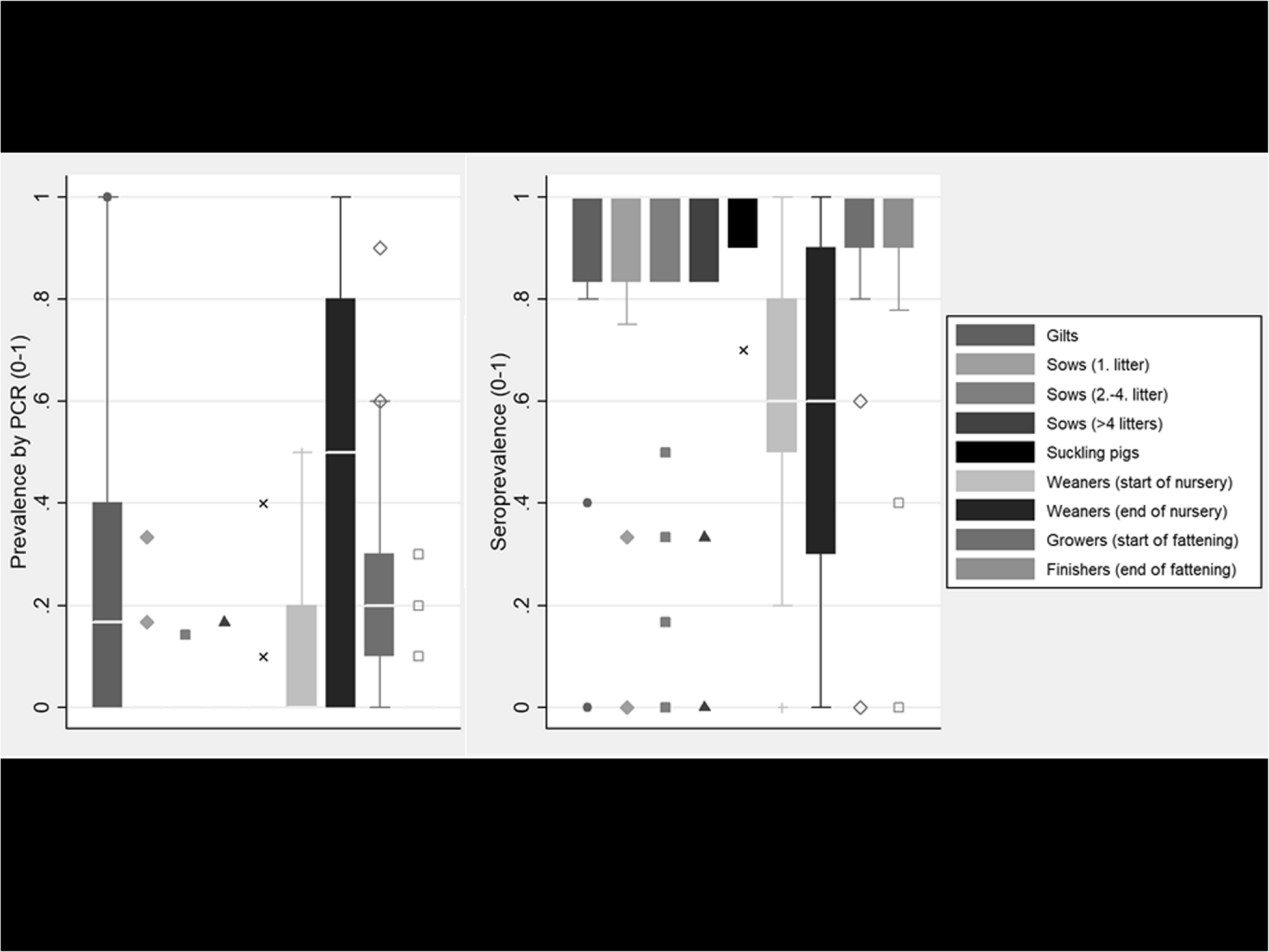
Laboratory results from PRRSV PCR and ELISA from 21 herds endemically infected with PRRSV. Legend: The boxes of the box-and-whisker plots indicate the interquartile range, the whiskers the 1.5-times interquartile range, and the single shapes below and above the outliers, separately for each age group

In 20 out of the 21 herds, PRRSV specific RNA (wild type virus) was detected by PCR in at least one of the blood samples. The mean prevalence of PRRSV independent of the age category was 14.2%. Samples from gilts, weaners and growers were more often positive than samples from pigs of any other age (Fig. 1). Weaners showed a trend of being more often positive for PRRSV, when they had been vaccinated against PRRSV during their suckling period (*P* = 0.059), whereas there was no association between vaccination status of pigs and their PRRSV-status in other age categories.

Serological testing was conducted on 1357 blood samples. This fraction does not include the samples from suckling pigs of 15 herds, where maternally derived antibodies were already expected because of sow vaccination.

In all herds, antibodies against PRRSV were detected in different age categories. The within-herd seroprevalence varied between 37.9 and 98.6% with a mean of 77.3%. Weaners at the start and at the end of their nursery period showed a lower seroprevalence when compared to other age categories (Fig. 1).

### Farm budget and costs of PRRS

**Fig. 2:**
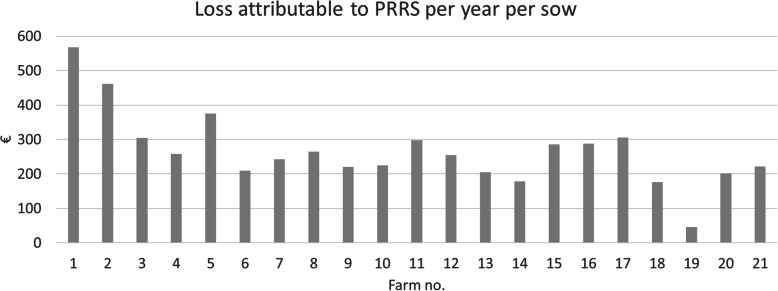
Loss per sow per year attributable to PRRS in 21 herds endemically infected with PRRSV

**Fig. 3:**
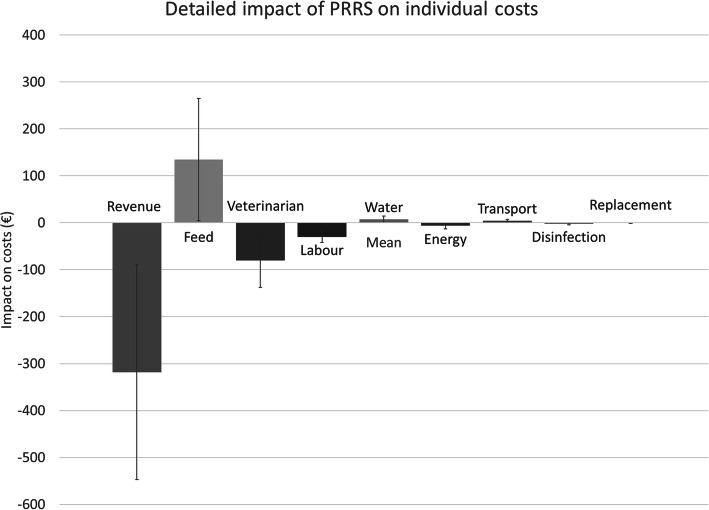
Detailed impact of PRRS on individual costs in 21 herds endemically infected with PRRSV. Legend: Negative bars indicate losses; positive bars indicate costs saved

Revenue and costs, resulting in the farm budget (= revenue - variable costs - fixed costs, indicating the profit) were calculated for each farm in its actual status and if it was negative for PRRSV (Table 4). The median farm budget across all 21 farms was − 31 € per sow and year, indicating a negative profit, as opposed to a simulated farm budget of 248 € if the farm had been PRRSV negative. The average costs of PRRS per year were estimated comparing the actual with the ‘negative’ farm budget for each farm (Table 5). The median loss per sow and year across all 21 farms was 250 € (range 46–568 €, visualized in Fig. 2), which corresponds to a median total loss per farm per year of almost 75,000 € attributed to endemic infection with PRRSV (range 16,540–306,395 €). In all cases, the biggest part of the total loss occurred in the fattening part, whereas the lowest financial impact was observed for the nursery part. In some cases, negative costs (i.e. a surplus) were obtained for the nursery, which can be explained by a significant amount of ‘costs saved’ and a low amount of ‘revenue foregone’ (data not shown). In contrast to this, ‘revenue foregone’ in the fattening part, because of not raising pigs on available fattening places, resulted in significant loss and turned total farm budgets in more than 50% of the 21 farms into deficit. The overall impact of ‘revenue foregone’, especially because of not selling the maximal possible number of fattening pigs and the increased veterinary costs, were outweighing the ‘costs saved’ by consuming less feed (Fig. 3).

**Table 4:** Median annual revenue, costs and resulting farm budget in Euro per sow for each herd, calculated for its actual status, and simulating that the same farm was negative for PRRSV

Farm no.	No. of sows	Status	Per sow per year (€)							
			Revenue finishers or piglets sold	Revenue sows sold	Replacement costs	Feeding costs	Veterinary costs	Other variable costs	Fixed costs	Farm budget
1	150	actual	4315	58	123	2968	238	136	974	**−67**
		*negative*	*5270*	*58*	*121*	*3439*	*200*	*128*	*939*	***501***
2	300	actual	3453	0	234	2157	258	244	952	**− 302**
		*negative*	*4216*	*90*	*233*	*2569*	*196*	*226*	*922*	***159***
3	178	actual	6635	90	137	3014	887	437	1117	**1133**
		*negative*	*6741*	*90*	*137*	*3004*	*715*	*438*	*1098*	***1438***
4	326	actual	7078	78	118	2614	166	466	1531	**2261**
		*negative*	*7262*	*78*	*118*	*2650*	*104*	*437*	*1512*	***2519***
5	198	actual	6660	95	181	2690	324	2536	2021	**− 997**
		*negative*	*7093*	*95*	*181*	*2831*	*282*	*2544*	*1972*	***− 622***
6	290	actual	4750	140	130	4256	356	362	897	**− 1111**
		*negative*	*4826*	*140*	*130*	*4227*	*286*	*346*	*880*	***− 902***
7	220	actual	4410	84	169	2704	174	176	1123	**149**
		*negative*	*4683*	*84*	*169*	*2833*	*95*	*172*	*1107*	***392***
8	510	actual	4988	81	164	3084	383	385	802	**253**
		*negative*	*5222*	*81*	*164*	*3185*	*270*	*388*	*778*	***518***
9	330	actual	4870	68	117	2368	280	285	1020	**868**
		*negative*	*5043*	*68*	*117*	*2405*	*215*	*283*	*1002*	***1088***
10	340	actual	4966	79	162	3402	265	274	942	**1**
		*negative*	*5124*	*79*	*162*	*3443*	*172*	*276*	*924*	***226***
11	225	actual	4814	77	157	3345	192	281	924	**−8**
		*negative*	*5237*	*77*	*157*	*3543*	*139*	*283*	*901*	***290***
12	1′200	actual	4625	99	192	3231	135	273	901	**−7**
		*negative*	*4942*	*99*	*191*	*3347*	*112*	*269*	*873*	***248***
13	270	actual	4467	88	174	3036	184	264	929	**−31**
		*negative*	*4806*	*88*	*174*	*3227*	*155*	*255*	*911*	***174***
14	340	actual	4380	70	168	3011	217	262	901	**−109**
		*negative*	*4488*	*70*	*168*	*3006*	*179*	*256*	*880*	***69***
15	350	actual	4239	72	134	2922	179	252	857	**−34**
		*negative*	*4657*	*72*	*134*	*3151*	*106*	*249*	*836*	***252***
16	200	actual	5120	59	129	3596	450	268	913	**− 178**
		*negative*	*5403*	*59*	*129*	*3730*	*325*	*272*	*896*	***110***
17	400	actual	4016	71	161	2802	173	236	822	**− 108**
		*negative*	*4642*	*71*	*161*	*3182*	*128*	*243*	*801*	***198***
18	400	actual	5043	99	192	3436	196	283	949	**85**
		*negative*	*5280*	*99*	*192*	*3566*	*143*	*284*	*933*	***261***
19	360	actual	4078	55	122	2791	175	246	872	**−73**
		*negative*	*4117*	*55*	*121*	*2811*	*159*	*241*	*867*	***−27***
20	450	actual	4401	46	128	2764	167	238	1000	**150**
		*negative*	*4640*	*46*	*128*	*2880*	*135*	*235*	*958*	***352***
21	408	actual	4320	76	150	2913	184	265	955	**−70**
		*negative*	*4615*	*76*	*148*	*3057*	*135*	*268*	*933*	***151***

**Table 5:** Median annual loss attributable to PRRS per sow for each herd, derived from the actual farm budget minus the ‘negative’ farm budget from Table 4, and annual loss for the herd in total and in the breeding, nursery and fattening part

Farm no.	Loss per year (€)				
	Per sow	Farm total	Breeding part	Nursery part^a^	Fattening part
1	568	85,263	8952	− 8480	84,791
2	461	138,454	24,132	−10,587	124,909
3	305	54,443	8886	25,502	20,055
4	258	84,085	19,958	10,991	53,136
5	375	74,181	16,375	− 7003	64,809
6	209	60,430	17,582	1199	41,649
7	243	53,396	16,370	1912	35,113
8	265	135,225	48,487	10,612	76,126
9	220	72,661	19,289	9889	43,483
10	225	76,447	32,409	1525	42,513
11	298	67,246	12,093	− 702	55,854
12	255	306,395	43,142	18,011	245,243
13	205	55,312	9945	4246	41,121
14	178	60,586	7443	16,757	36,386
15	286	100,211	27,314	− 6462	79,360
16	288	57,528	18,000	8853	30,674
17	306	122,393	29,985	−22,956	115,364
18	176	70,324	22,298	6036	41,991
19	46	16,540	7846	− 680	9375
20	202	90,503	16,679	8368	65,456
21	221	90,371	21,618	− 2253	71,006
**Median**	**255**	**74,181**	**18,000**	**1912**	**53,136**

^a^Negative values mean that the herd did not see a loss but saved costs in the nursery part

The impact of PRRS reduced farm profits by − 19.1% on average (SD 10) and − 41% in the worst case (the minimum was 0%). This translates into significant loss of economic and/or normal profit, which accounts for revenue and costs including opportunity costs.

## Discussion

The present study aimed to apply and evaluate the practical applicability of a PRRS economic calculator for veterinarians under field conditions by estimating losses due to endemic PRRSV infection in 21 pig farms in Germany. This is, to the authors’ knowledge, the first study to apply a simulation tool in order to systematically calculate the cost of PRRS endemic infection for various different farms in a standardized and thus comparable way. Most calculations available elsewhere to date are either anecdotal, non-standardized reports from single farms, calculate the cost for the epidemic period only, and/or date from several years ago and thus do not reflect the current economic situation.

Results from this study indicate a significant loss due to PRRSV infection across farms. All farms in the study experienced a loss, and the median was 255 Euro per sow and year, ranging from 46 to 568 Euro. These figures are very similar to those obtained from the same model after applying it to different hypothetical disease scenarios, indicating losses between 126 Euro and 650 Euro per sow and year depending on disease severity [20]. The fact that in the present study no association between the amount of PRRS-attributable losses in a herd and its laboratory detection rate and degree of clinical affectedness (expressed as alterations in health and performance parameters) could be seen, can be explained by the very diverse farm and general cost structures of herds enrolled in this study. This is substantiated by the very diverse estimates of farm budgets for these farms if they had been PRRSV-negative.

These resulting losses per sow and year are within the range of figures found elsewhere. In a Spanish report dating from 2013, losses during 6 months following an outbreak were estimated as being 200 US$ per sow for a farrow-to-finish farm and 122 US$ per sow for a breeding farm [24]. Another report from 2015 estimated costs of PRRS for chronically infected herds in UK to be 135 Euro per sow and year, whereas for the Netherlands and Denmark with 100 € per sow and year in acutely affected herds, estimations were somewhat lower [25]. Figures available for Germany, a projection of costs of PRRS on the whole German sow inventory (not only PRRSV infected herds), indicated an overall average of 150 Euro per sow and year [26].

Similar as in previous studies [4], the biggest loss in all study farms arose in the fattening part due to the huge revenue foregone because fewer pigs were sold. The second highest impact of PRRS on the cost structure was seen on the costs for feed. All except three study farms experienced lower feeding costs than in the negative baseline scenario, because there were fewer animals to be fed. This was especially obvious in the nursery part where some farms even saved costs due to lower feed costs for fewer nursery pigs. The third highest impact of PRRS was seen on veterinary costs, where all farms had higher costs than in the negative baseline scenario. All other revenue and cost items were less affected by PRRS.

When interpreting the results it has to be kept in mind that the number of farms enrolled in the study was limited and not randomly chosen, since inclusion depended on the operation manager’s willingness to participate and provide the required data. Farms were selected upon their PRRS history as reported by the herd-attending veterinarian. To substantiate that a study farm really suffered from PRRSV (endemic) infection and this could be assumed to be the main reason for financial losses, an extensive sampling for PRRSV detection (direct and indirect) was carried out on each farm. A number of roughly 60 to 70 samples per herd is considered to be sufficient to reveal the true PRRSV status of a herd. This sampling showed that indeed all study farms were exposed to PRRSV, with antibodies found in all, and wild type virus detected in all but one farm, substantiating their enduring PRRSV infection. This, together with the observed alterations in various health and performance parameters typical for PRRSV infection and not explicable by other influencing factors (e.g. management factors), make it seem very likely that estimated losses can be attributed to PRRS.

Of course, the calculation of losses due to PRRS in this economic calculator remains a simulation and the resulting figures hypothetical. A before - after comparison of farm budgets for these herds is not possible since these herds have been PRRSV infected for an unknown and certainly longer time period. Furthermore, for some of the farms the calculation might be imprecise since in the absence of available data on certain costs, standard (i.e. default) values had to be used for these cost items.

In addition, the calculation of costs of PRRS was done by deriving and comparing a hypothetical farm budget if the herd was PRRSV negative from/to the actually calculated farm budget of the respective herd. For this, several assumptions had to be made on how a ‘PRRSV negative’ farm would look like in terms of reference i.e. ‘normal’ values for health and performance parameters. Since it was not possible for all parameters to find average values specifically from confirmed PRRSV negative herds, average values from country-wide production performance data were used. These most certainly also include PRRSV positive farms. That means, the true values for PRRSV negative herds are probably even higher or better than the baseline values we used for comparison in the model. The consequence is that the model tends to underestimate the true loss and gives a conservative estimate of the costs of PRRS. Apart from that, the median simulated farm budget, i.e. profit, for a PRRSV negative farm of 260 Euro per sow and year is well in line with average farm budgets reported for German pig farms in recent years: between 100 and 300 Euro per sow and year, depending on source and year [27–29] This shows that the calculator as such renders a valid estimation of general farm economics.

Detection rates of PRRSV and antibodies within study herds were well in range of those in other studies for endemically infected herds, with generally high seroprevalences and similar age dependent distributions [30, 31]. This suggests that the herds in this study are a well representative of endemically PRRSV infected herds in general, and that the calculated PRRS-attributable losses can serve as general estimate of average losses for endemically infected herds in Germany. Since PRRSV herd prevalence in Germany is considered high, estimated around 50 to 75% [25, 32] and studies indicate 30% instable herds (i.e. with virus detection in suckling pigs), this suggests that the calculated losses can be expected to occur in a big part of herds in Germany. And the situation is similar in other countries where studies estimated herd prevalence of similar magnitude, like 20–60% in Danish studies [33, 34], or more than 50% in a French study [31].

Therefore, the obtained losses, although only an estimate and derived from a simulation, give a good hint of the economic scale of the damage that PRRS is causing for the (German) pig industry. It emphasizes that even in endemically infected farms, farmers face a non-negligible economic damage due to PRRS, and that they can profit from a concerted PRRS control in their farm.

## Conclusions

The calculated losses give a good hint of the economic damage due to PRRS for the pig industry. Even in endemically infected farms, farmers face a non-negligible damage and profit from a concerted PRRS control. The economic calculator has proven itself in the field to render a valid, conservative estimation of losses due to PRRS in endemically infected farms. The obtained costs per farm will serve as basis for the calculator to indicate the best intervention strategy for each farm. Aim of a following study will be to apply this strategy and finally evaluate its success for each study farm.

## Methods

### PRRS herd-level cost model

The model used in the present study to estimate the cost of PRRS in an infected farm has been established earlier by Nathues and others [20]. Consisting of three parts (breeding, nursery, fattening), the economic model can simulate production of the following different farm types: 1) breeding farms with sale of piglets at weaning; 2) breeding farms with sale of nursery pigs; 3) nursery farms; 4) fattening farms; 5) farrow-to-finish farms. Furthermore, it can be customized to farm-specific settings, production performance, disease parameters and prices, since it should serve as a decision making tool for farmers and veterinarians at individual-farm-level. 1) In a first step, the model calculates the current farm budget for the farm in question. The farm budget is defined as the farm’s revenue minus its variable and fixed costs, and indicates the farm’s profit. 2) In a next step, the model simulates a baseline farm budget for this farm, assuming that this farm was negative for PRRSV, by correcting for the changes in health and production performance attributable to PRRS. For this, all production and health parameters commonly affected by PRRS (the parameters in italic in Tables 1, 2 and 3) are set to a baseline value that could be expected in an average, presumably healthy and PRRSV negative, farm. This means, wherever the baseline value for a parameter is better than the farm’s actual value, the model uses this baseline value instead of the farm’s actual value. This set of parameters as well as the corresponding baseline values were retrieved from country-specific literature on production performance. Since not for all parameters average values specifically for PRRSV negative herds were available, average values from the country’s general pig population were used as a proxy [35, 36]. These baseline values are: a return-to-estrus rate of ≤10%; an abortion rate of ≤2%; an average number of piglets born alive (depending on genetics, > 12.7 on average); a pre-weaning mortality of ≤11%; a weight at weaning of ≥6 kg (with a 4 week suckling period); a mortality in weaners of ≤3% and fatteners of ≤1.5%; and 0% PRRS morbidity in weaners and fatteners. Further details on this are available in the paper describing the model [20]. 3) Finally, the simulated farm budget from the negative baseline scenario is compared to the actual farm budget of the farm in question. The resulting difference is then the estimated average farm specific loss, calculated on a yearly basis, with the underlying assumption that PRRSV is the cause of the changes in productivity. The simulation tool is available via the “PRRS integrated solutions” website and smartphone app (www.integrated-prrs-solutions.com; Merck Animal Health, New Jersey, United States of America).

### Herd enrolment and selection criteria

The study was carried out between April 2017 and November 2017. For demonstrating the application of the model, in total 21 farms were included, 19 one-site production systems and 2 two-site production systems with a 1:1 supply relationship (1 (one) piglet producing farm supplying 1 (one) fattening farm, which does not receive piglets from any other source). Participating farms were designated from veterinary practitioners from all over Germany matching the following criteria: herds should suffer from an endemic PRRSV infection, meaning that the herd-attending veterinarian had first and repeatedly diagnosed PRRSV infection at least 12 months prior to the study. Furthermore, they should not have epidemics of other primary infectious diseases (i.e. no clinical or laboratory confirmed diagnosis by the herd-attending veterinarian) or major flaws or changes in farm management within the last 12 months. Lastly, only farms with a minimal herd size of 100 sows and the ability to provide necessary information to run the economic disease model were included. Vaccination against PRRSV was no exclusion criterion. In detail, 14 farms were in Lower Saxony and 5 in the adjacent North of North Rhine-Westphalia, both areas with high pig density. Two of the enrolled herds were in less pig dense areas (Baden-Wuerttemberg, Thuringia).

### Data collection

In advance to the sampling period, a standardized questionnaire following the structure of the calculator was developed. Data on health and production performance, farm management and environment of the PRRS affected herds were collected during a one-time farm visit from the operations manager. All questionnaires were filled by the same investigator in order to prevent any observer variation.

### Sample size and collection

In the course of routine diagnostics and to evaluate the PRRSV status of the farm according to Holtkamp [37] a modified protocol was applied, where blood samples from different age groups were taken during the farm visit. The sample size for ‘estimation of percentage’ (i.e. prevalence estimation) was calculated based on an average number of 350 sows per herd, 25% expected prevalence and an accepted error of 20% (adjusted sample size: 18). For estimating the frequency of PRRSV infection in the offspring (infinite population size), an expected prevalence of 50% and accepted error of 20% were considered (adjusted sample size: 24). The samples were collected from the following age and production categories: incoming/mature gilts (*n* = 5), 18 sows (parity 1 (*n* = 6), parity 2–4 (*n* = 6), parity > 4 (*n* = 6), 10 suckling pigs (approx. 3 weeks of age), 10 weaners at the start of nursery (approx. 6 weeks of age), 10 weaners at the end of nursery (approx. 9 weeks of age), 10 growers (approx. 16 weeks of age) and 10 finishers (approx. 22 weeks of age). Within the different age categories, animals were selected by chance after marking individual pigs with color spray during a random walk through the pens. Pigs up to 6 weeks of age were restrained on the lap of a helping person and blood was collected by puncture of the *Vena cava cranialis.* Pigs older than 6 weeks were restrained by snare and sample collection was performed using *Vena jugularis externa.* Samples were stored (7 °C, at least 2 h) and after centrifugation (1′000 G, 10 min) serum was transferred into reaction tubes and stored at −20 °C until shipment to lab.

### Laboratory investigation

All blood samples were sent to the laboratory of Intervet BV in Boxmeer, Netherlands. They were tested for PRRSV antibodies using HerdCheck PRRS X3 ELISA kit manufactured by IDEXX. Besides a PRRSV PCR was executed with the virotype PRRS RT-PCR kit of Indical. Vaccine virus was detected by DV-PCR and, if present, the sample was considered ‘negative for PRRSV (wild type)’. All tests were performed and evaluated according to manufacturers’ instructions.

### Data analysis

All information from the questionnaires was stored in Excel 2016 (Microsoft Corporation, Redmont, Washington, USA) that simultaneously enables running the model, which was developed in Excel 2010 and is utilizing @RISK software for Excel version 6.3.1 (Palisade Corporation, Newfield, New York, USA) in order to account for probability distributions of input variables. The results from the epidemiological characterization of the 21 farms and the outputs from the economic calculator after 10′000 iterations in @RISK software were summarized and submitted to descriptive statistics using STATA/IC 12.0 for Windows [64-bit ×86–64] (StataCorp LP, Texas, USA).

## Data Availability

The questionnaire and dataset used are available from the corresponding author on reasonable request.

## Abbreviations

Max: Maximum
Min: Minimum
PRRS: Porcine Reproductive and Respiratory Syndrome
PRRSV: Porcine Reproductive and Respiratory Syndrome Virus
SD: Standard deviation
